# Fabrication Technology and Characteristics Research of a Monolithically-Integrated 2D Magnetic Field Sensor Based on Silicon Magnetic Sensitive Transistors

**DOI:** 10.3390/s18082551

**Published:** 2018-08-04

**Authors:** Xiaofeng Zhao, Chenchen Jin, Qi Deng, Meiwei Lv, Dianzhong Wen

**Affiliations:** School of Electronics Engineering, Heilongjiang University, Harbin 150080, China; 2161330@s.hlju.edu.cn (C.J.); 2151254@s.hlju.edu.cn (Q.D.); 2171251@s.hlju.edu.cn (M.L.); wendianzhong@hlju.edu.cn (D.W.)

**Keywords:** two-dimensional magnetic field sensor, silicon magnetic sensitive transistor, monolithic integration, difference structure, MEMS technology

## Abstract

A monolithically-integrated two-dimensional (2D) magnetic field sensor consisting of two difference structures (DSІ and DSII) is proposed in this paper. The DSІ and DSII are composed of four silicon magnetic sensitive transistors (SMST1, SMST2, SMST3 and SMST4) and four collector load resistors (*R_L_*_1_, *R_L_*_2_, *R_L_*_3_ and *R_L_*_4_). Based on the magnetic sensitive principle of SMST, the integrated difference structure can detect magnetic fields’ component (*B_x_* and *B_y_*) along the *x*-axis and *y*-axis, respectively. By adopting micro-electromechanical systems (MEMS) and packaging technology, the chips were fabricated on a p-type <100> orientation silicon wafer with high resistivity and were packaged on printed circuit boards (PCBs). At room temperature, when the *V*_CE_ = 5.0 V and *I_B_* = 8.0 mA, the magnetic sensitivities (*S_xx_* and *S_yy_*) along the *x*-axis and the *y*-axis were 223 mV/T and 218 mV/T, respectively. The results show that the proposed sensor can not only detect the 2D magnetic field vector (***B***) in the *xy* plane, but also that *S_xx_* and *S_yy_* exhibit good uniformity.

## 1. Introduction

In this paper, a monolithically-integrated 2D magnetic field sensor was designed and fabricated by MEMS technology. In order to improve the magnetic sensitivity and uniformity, we integrated two difference structures (DSI; and DSII) with four SMSTs and four collector resistors as a magnetic sensitive structure along the direction of the *x*-axis and *y*-axis, respectively. On this basis, theoretical analysis shows the effect of the magnetic field component (*B_x_* and *B_y_*) on the output voltage of the proposed sensor. Meanwhile, the *I*_C_-*V*_CE_ characteristics of SMSTs and the magnetic sensitivity of DSI; and DSII were tested, and we studied the uniformity and cross interference of the magnetic sensitivity (*S_xx_* and *S_yy_*).

At present, magnetic field sensors include the Hall element, giant magnetoresistance (GMR), tunneling magnetoresistance (TMR), magnetic sensitive diodes (MSD), silicon magnetic sensitive transistors (SMST), and so on [1,2,3,4]. With the development of microelectromechanical systems (MEMS) technology, magnetic field sensors have achieved a three-dimensional structure, miniaturization and integration and have a wide range of applications, such as industrial, military, aerospace and other areas [5,6,7,8]. In 2012, Chih-Ping Yu et al. proposed a two-dimensional difference folded Hall device, which integrated the lateral magnetic transistor (LMT) with the magnetoresistor (MR) [9]. When the bias current was 100 mA and the supply voltage was 2.7 V, the results show that the optimum magnetosensitivity (*S_RI_*), optimum sensitivity (*S*), minimum nonlinearity error (NLE) and minimum offset were 0.385 V/(A·T), 9.564 mV/T, 4.03% and 18.85 mV, respectively. In 2013, Guo-Ming Sung et al. proposed a vertical Hall device (VHD) based on the combined magnetic effects between a bulk magnetotransistor (BMT), a vertical magnetoresistor (VMR) and a vertical magnetotransistor (VMT), which was sensitive to the magnetic induction in the plane [10]. The p-substrate was used to enhance the magnetosensitivity of BMT. The maximum supply-current-related magnetic sensitivity (*S_I_*), maximum supply-voltage-related magnetic sensitivity (*S_V_*) and minimum mean NLE were 1.92 V/(A·T), 42.65 mV/(V·T) and 2.11%, respectively. In 2015, Haiyun Huang et al. proposed a monolithic complementary metal oxide semiconductor (CMOS) magnetic Hall sensor with high sensitivity and linearity characteristics [11]. The current-related sensitivity (*S_I_*) and the voltage related sensitivity (*S_V_*) achieved were 250 V/(A·T) at a 1-mA biasing current and 0.034 V/(V·T) at a bias voltage of 3 V. In 2017, a packaging integrated 2D magnetic field sensor was proposed, which consisted of four discrete SMSTs (the area of an SMST is 2.0 × 2.0 mm^2^) and four load resistors [12]. When the *V*_CE_ = 10.0 V and *I_B_* = 6.0 mA, the magnetic sensitivities of the sensor along the *x*-axis and *y*-axis were 366.0 mV/T and 365.0 mV/T, respectively. By combining or integrating multiple magnetic sensitive devices, the 2D magnetic field (*B_x_* and *B_y_*) in the *xy* plane can be measured. Based on the above references, the characteristics of sensitivity, uniformity and cross interference have been thoroughly studied. These properties have been improved by technological improvement and structural optimization. Summing up, monolithic integration has achieved greater improvement in uniformity and reduced cross interference.

## 2. Basic Structure and Working Principle

### 2.1. Basics Structure

**Figure 1:**
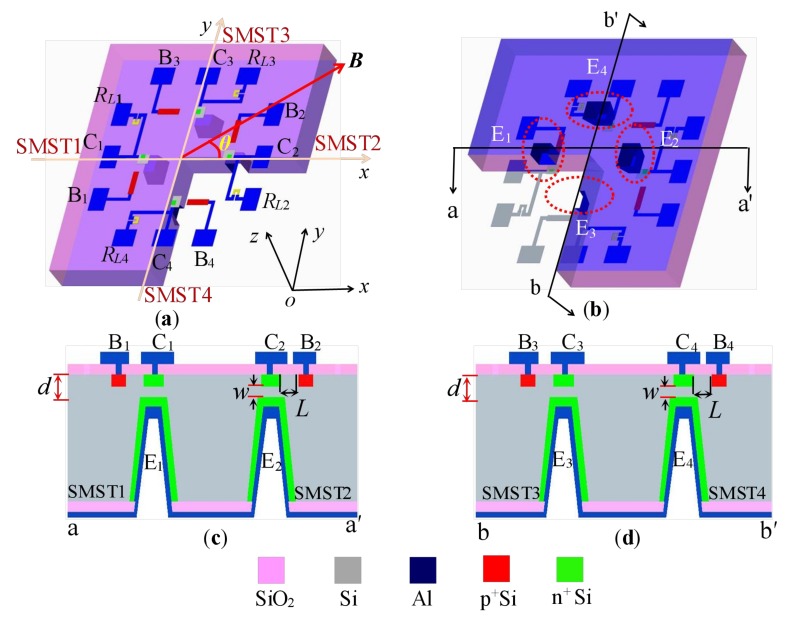
The basic structure of the monolithically-integrated 2D magnetic field sensor: (**a**) front of the basic structure; (**b**) back of the basic structure; (**c**) cross-section along aa′; (**d**) cross-section along bb′. SMST, silicon magnetic sensitive transistor.

Figure 1 shows the cubic structure of the monolithically-integrated two-dimensional (2D) magnetic field sensor, which is made up of two difference structures. The DSІ consists of two SMSTs (SMST1 and SMST2) and two collector load resistors (*R_L_*_1_ and *R_L_*_2_) that can detect the magnetic field (*B_x_*) along *x*-axis, and two SMSTs with the opposite magnetic sensitive direction are placed symmetrically along the *x*-axis in *xy* plane. The DSII is composed of two SMSTs (SMST3 and SMST4) and two collector load resistors (*R_L_*_3_ and *R_L_*_4_) that exhibit the opposite magnetic sensitive direction along the *y*-axis. As shown in Figure 1a,b, four bases (B_1_, B_2_, B_3_, B_4_) and four collectors (C_1_, C_2_, C_3_, C_4_) are designed on the chip surface, and four emitters (E_1_, E_2_, E_3_, E_4_) are designed on the back of the chip by MEMS technology. Figure 1c,d shows the cross-section of the proposed sensor along the aa′ and bb′ directions, where *L* is the length of the base region, *w* is the width of the base region and *w* > *L*_D_ (*L*_D_ is the carrier diffusion length), *d* is the thickness of the silicon membranes and *θ* is the angle between the external magnetic field vector (***B***) and the +*x*-axis of the chip surface. As illustrated in Figure 1a, the external magnetic field along the +*x*-axis (or +*y*-axis) is defined as a positive magnetic field *B_x_* > 0 T (or *B_y_* > 0 T), and the opposite direction is defined as the reverse magnetic field *B_x_* < 0 T (or *B_y_* < 0 T).

### 2.2. Working Principle

**Figure 2:**
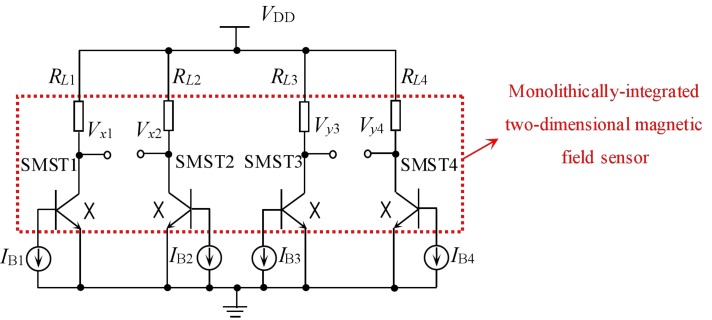
Equivalent circuit of the monolithically-integrated 2D magnetic field sensor.

Figure 2 presents an equivalent circuit of the proposed sensor, where the red dashed box consists of four SMSTs and four collector load resistors for the monolithically-integrated chip, in which *R_L_*_1_, *R_L_*_2_, *R_L_*_3_ and *R_L_*_4_ are the collector load resistors of four SMSTs, respectively. One end of *R_L_*_1_, *R_L_*_2_, *R_L_*_3_ and *R_L_*_4_ is connected to *C*_1_, *C*_2_, *C*_3_ and *C*_4_ of the SMSTs, respectively, and the other ends of *R_L_*_1_, *R_L_*_2_, *R_L_*_3_ and *R_L_*_4_ are connected to the supply power. *V_x_*_1_, *V_x_*_2_, *V_y_*_3_ and *V_y_*_4_ are the collector output voltages for SMST1, SMST2, SMST3 and SMST4, respectively. *I_B_*_1_, *I_B_*_2_, *I_B_*_3_ and *I_B_*_4_ are current sources that provide the base injection currents for *B*_1_, *B*_2_, *B*_3_ and *B*_4_, respectively. *V*_DD_ is the supply voltage. The four emitters of SMSTs are commonly grounded.

**Figure 3:**
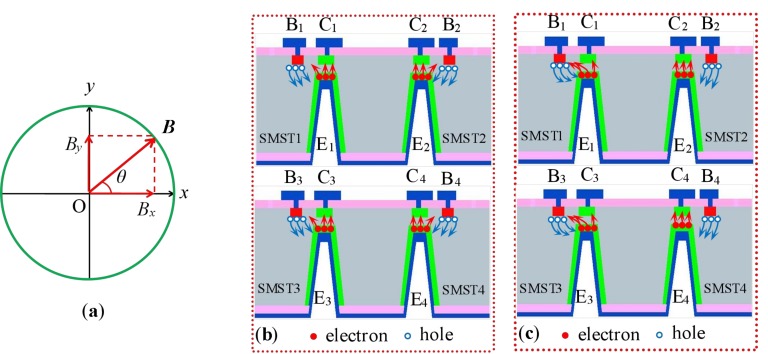
Working principle of the monolithically-integrated 2D magnetic field sensor: (**a**) magnetic field vector in the *xy* plane; (**b**) *B* = 0 T; (**c**) *B* ≠ 0 T.

As shown in Figure 3a, ***B*** is the magnetic field vector in the *xy* plane, where *θ* is the angle between ***B*** and the +*x*-axis. The magnetic field components *B_x_* and *B_y_* along the *x*-axis and the *y*-axis are expressed as:

(1) $$\left\{ \begin{array}{l} B_{x}=B\cos\theta \\ B_{y}=B\sin\theta \end{array}\right.$$

where *B* is magnetic induction intensity.

When *B* = 0 T, in ideal conditions, the carriers (electrons and holes) are not affected by the Lorentz force and are not deflected, so the collector currents of the four SMSTs are equal (*I_C_*_1_ = *I_C_*_2_ = *I_C_*_3_ = *I_C_*_4_ = *I_C_*_0_). As shown in Figure 3b, under the condition of *R_L_*_1_ = *R_L_*_2_ = *R_L_*_3_ = *R_L_*_4_ = *R*_0_, the output voltages (*V_x_* and *V_y_*) of the proposed sensor are as follows:

(2) $$\left\{ \begin{array}{l} V_{x}=V_{x1}-V_{x2}=I_{C2}\cdot R_{L2}-I_{C1}\cdot R_{L1}=0\\ V_{y}=V_{y3}-V_{y4}=I_{C4}\cdot R_{L4}-I_{C3}\cdot R_{L3}=0 \end{array}\right.$$

where *I_C_*_1_, *I_C_*_2_, *I_C_*_3_ and *I_C_*_4_ are the collector currents of four SMSTs, respectively.

As shown in Figure 3c, when *B* ≠ 0 T, under the action of *B_x_* (*B_x_* > 0), the carrier of SMST1 (SMST2) is deflected by the Lorentz force, leading to a decrease (increase) in the number of carriers collected by the collector region, so that *I_C_*_1_ decreases (*I_C_*_2_ increases). Similarly, under the action of *B_y_* (*B_y_* > 0), *I_C_*_3_ and *I_C_*_4_ of SMST3 and SMST4 are decreased and increased, respectively. *V_x_* and *V_y_* are expressed as:

(3) $$\left\{ \begin{array}{l} V_{x}=V_{x1}-V_{x2}=(I_{C1}+\Delta I_{C2})\cdot R_{L2}-(I_{C1}+\Delta I_{C1})\cdot R_{L1}=\Delta V_{x2}-\Delta V_{x1}\\ V_{y}=V_{y3}-V_{y4}=(I_{C4}+\Delta I_{C4})\cdot R_{L4}-(I_{C3}+\Delta I_{C3})\cdot R_{L3}=\Delta V_{y4}-\Delta V_{y3} \end{array}\right.$$

where ∆*V_x_*_1_, ∆*V_x_*_2_, ∆*V_y_*_3_ and ∆*V_y_*_4_ are the variations of the collector output voltages of SMST1, SMST2, SMST3 and SMST4, respectively. ∆*I_C_*_1_, ∆*I_C_*_2_, ∆*I_C_*_3_ and ∆*I_C_*_4_ are the variations of collector currents of four SMSTs, respectively.

According to the definition of the magnetic sensitivities for the proposed sensor, the voltage magnetic sensitivities (*S_xx_* and *S_yy_*) along the *x*-axis and *y*-axis can be given:

(4) $$\left\{ \begin{array}{l} S_{xx}=\frac{\left|V_{x}\right|}{B_{x}}=\frac{\left|\Delta V_{x2}-\Delta V_{x1}\right|}{B_{x}}=\left|S_{x1}+S_{x2}\right|\\ S_{yy}=\frac{\left|V_{y}\right|}{B_{y}}=\frac{\left|\Delta V_{y4}-\Delta V_{y2}\right|}{B_{y}}=\left|S_{y3}+S_{y4}\right| \end{array}\right.$$

where *S_x_*_1_, *S_x_*_2_, *S_y_*_3_ and *S_y_*_4_ are the voltage magnetic sensitivities of SMST1, SMST2, SMST3 and SMST4, respectively.

Through theoretical analysis, magnetic field sensor with the difference structure of SMSTs can improve the magnetic sensitivity. On the basis of Equations (1)–(3), *V_x_* and *V_y_* can be obtained:

(5) $$\left(\begin{array}{l} V_{x}\\ V_{y} \end{array}\right)=\left(\begin{array}{cc} S_{xx} & S_{xy}\\ S_{yx} & S_{yy} \end{array}\right)\left(\begin{array}{l} B_{x}\\ B_{y} \end{array}\right)$$

where *S_xy_* and *S_yx_* are the cross magnetic sensitivities of the sensors. Under ideal conditions, *S_xx_* = *S_yy_* = *S* and *S_xy_* = *S_yx_* = 0, the *S_xx_* and *S_yy_* have a good uniformity. Then, the output voltage (*V*_out_) can be denoted by:

(6) $$V_{\mathrm{out}}\;\sqrt{V_{x}{}^{2}+V_{y}{}^{2}}\;=\;\sqrt{S_{xx}{}^{2}B^{2}\;\cos^{2}\;\theta +S_{yy}{}^{2}B^{2}\;\sin^{2}\;\theta }\;=\;S\cdot B$$

From Equation (5), we can see that DSІ and DSII are used to achieve the measurement of the magnetic field components *B_x_* and *B_y_* in the *xy* plane, respectively. In addition, the *V*_out_ of the proposed sensor under the action of ***B*** is calculated by Equation (6). When *B* is a certain value, under the ideal case, the magnetic sensitivity has good uniformity, and *V*_out_ does not change with *θ*. After theoretical analysis, the proposed sensor has 2D magnetic sensitive characteristics and can achieve the geographic orientation measurement in plane at the same time.

## 3. Fabrication Technology

**Figure 4:**
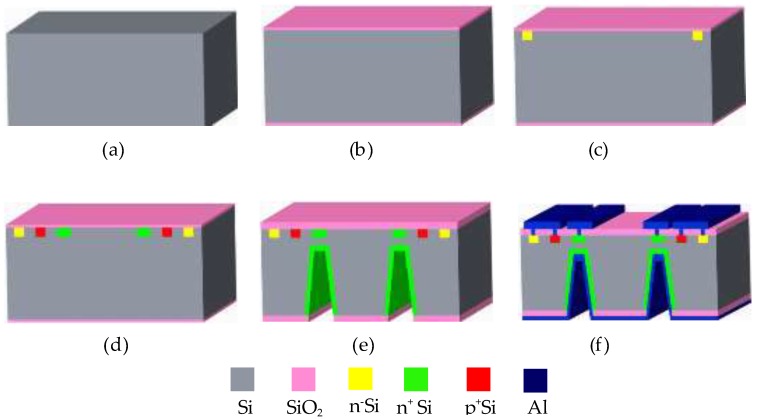
Main fabrication technology process of the proposed sensor chip: (**a**) cleaning the silicon wafer; (**b**) growing thin oxide; (**c**) lithography and ion implantation form load resistors and collector regions; (**d**) making the base regions by lithography and ion implantation; (**e**) lithography and ion implantation to form four emitter regions; (**f**) lithography and deposition of Al electrodes and metallization to form an ohmic contact.

Based on the basic structure of the proposed sensor, the chip of the sensor was fabricated on a double-sided polished p-type <100> orientation silicon wafer with high resistivity. Figure 4 shows the main fabrication process of the sensor. The process steps are as follows: (a) cleaning the silicon wafer by using the Radio Corporation of America (RCA) standard cleaning method; (b) the first oxidation, growing the SiO_2_ layer by the thermal oxidation method, with a thickness of 30 nm–40 nm; (c) etching windows of the collector load resistor, injecting phosphorus ions by the ion implantation process and forming collector load resistors; (d) etching windows of the collector region, shaping the n^+^ regions adopted phosphorus ion implantation process, photoetching base region windows, concentrated boron injection to form p^+^ regions and removing the SiO_2_ layer of the upper surface, after re-growth of the SiO_2_ layer (thickness of 500 nm); (e) lithography emitter region windows, etching silicon cups by inductively-coupled plasma (ICP) and injecting phosphorus ions to form emitter junctions of four SMSTs; (f) photolithography of the lead hole; the Al layer was deposited on the surface of silicon wafer by the magnetron sputtering method, etching metal Al to form four collectors (*C*_1_, *C*_2_, *C*_3_ and *C*_4_), four bases (*B*_1_, *B*_2_, *B*_3_ and *B*_4_) and the interconnect wire on the surface of the chip, depositing the Al electrode on the back of the wafer to form a common emitter and metallization to form an ohmic contact (30 min at 420 °C).

**Figure 5:**
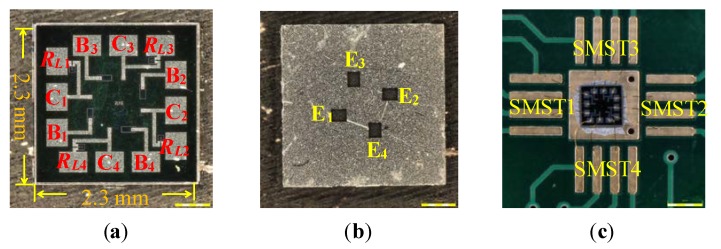
Photograph of the chip of the monolithically-integrated 2D magnetic field sensor: (**a**) front of the chip; (**b**) back of the chip; (**c**) photograph of the packaging of the monolithically-integrated chip.

Figure 5a shows the front photograph of the fabricated sensor. The area of the chip is 2.3 mm × 2.3 mm. *B*_1_, *B*_2_, *B*_3_ and *B*_4_ are the bases, *C*_1_, *C*_2_, *C*_3_ and *C*_4_ are the collectors and *R_L_*_1_, *R_L_*_2_, *R_L_*_3_ and *R_L_*_4_ are the collector load resistors of the four SMSTs. As shown in Figure 5b, *E*_1_, *E*_2_, *E*_3_ and *E*_4_ are the emitters of the four SMSTs, respectively. They form the common emitters. The chip was packaged on printed circuit boards (PCBs) using internal lead bonding technology, and a photograph is shown in Figure 5c.

## 4. Characteristics of the 2D Magnetic Field Sensor

### 4.1. Testing System

**Figure 6:**
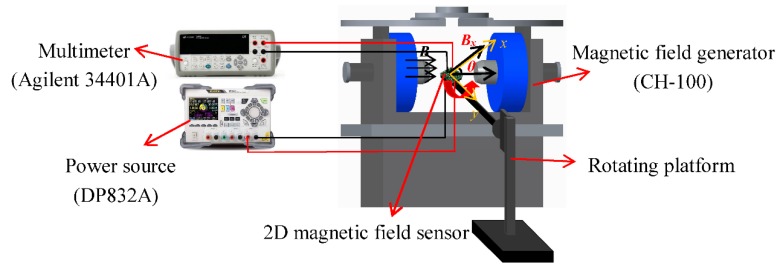
The test system of the monolithically-integrated 2D magnetic field sensor.

Figure 6 shows the testing system of the proposed sensor, which consists of a magnetic field generator (CH-100, Cuihai, Beijing, China), a multi-meter (Agilent 34401A, Agilent, Santa Clara, CA, USA), a programmable linear DC power source (DP832A, RIGOL, Beijng, China) and a rotating platform. At room temperature, we studied the *I*_C_-*V*_CE_ characteristics of the SMSTs using a semiconductor characteristic analysis system (KEITHLEY 4200, Keithley, Cleveland, OH, USA). On this basis, the magnetic sensitive characteristics for the 2D magnetic field sensor were tested using the testing system. In the testing process, the chip of sensor was placed on the surface of a rotating platform. By adopting a rotating platform with a programmable motor, we adjusted the angle (*θ*) between the constant magnetic field vector (***B***) and the magnetic sensitive direction for the *x*-axis sensor. According to Equations (1)–(3), when *B* is constant, the relationship curves between the output voltage (*V_x_* and *V_y_*) and the *θ* are tested.

### 4.2. I_C_-V_CE_ Characteristics of the SMSTs

**Figure 7:**
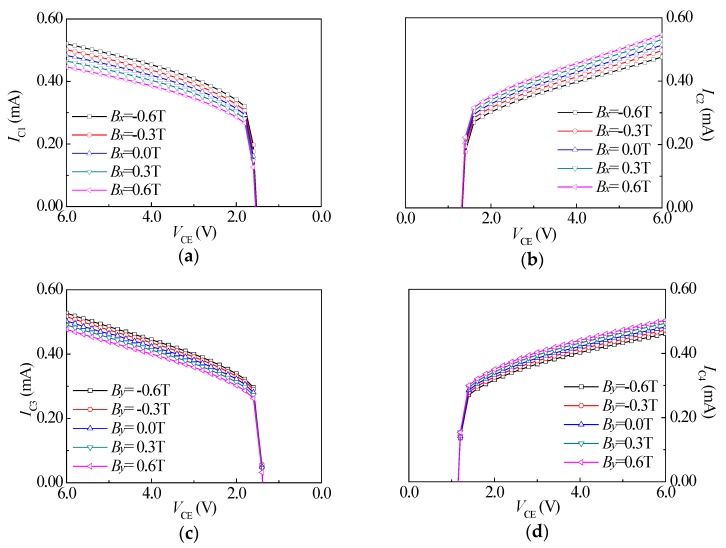
The *I*_C_-*V*_CE_ characteristic curves of the four SMSTs under different magnetic fields: (**a**) SMST1; (**b**) SMST2; (**c**) SMST3; (**d**) SMST4.

In a range of supply collector voltage of 0 V–6.0 V (the test step is 0.2 V) and a base injection current (*I_B_*) of 8.0 mA, the effect of the external magnetic field (*B* = 0 T, *B* = ±0.3 T and *B* = ±0.6 T) on the *I*_C_-*V*_CE_ characteristics of SMSTs was researched. When a magnetic field is applied on the chip along the *x*-axis direction, Figure 7a,b shows the *I*_C_-*V*_CE_ characteristic curves of SMST1 and SMST2 at different *B_x_* (and *B_y_* = 0 T). We can see that SMST1 and SMST2 have the opposite magnetic sensitive characteristics. When *V*_CE_ > 1.8 V, the *I_C_*_1_ of SMST1 keeps increasing in the whole magnetic field range from the positive magnetic field to the negative magnetic field. However, in the same condition, the *I_C_*_2_ of SMST2 keeps decreasing. It was also shown that when *V*_CE_ was a fixed value and *B_x_* > 0 T, the *I_C_*_1_ of SMST1 decreased with *B_x_* and the *I_C_*_2_ of SMST2 increased with *B_x_*. When *B_x_ <* 0 T, the *I_C_*_1_ of SMST1 increased with *B_x_* and the *I_C_*_2_ of SMST2 decreased with *B_x_*. Furthermore, when a magnetic field *B* was applied along the *y*-axis direction (and *B_x_* = 0 T), the *I*_C_-*V*_CE_ characteristic curves of SMST3 and SMST4 were as shown in Figure 7c,d. Similarly, SMST3 and SMST4 also had the opposite magnetic sensitive characteristics. Moreover, the *I*_C_-*V*_CE_ characteristic curves of SMST3 and SMST4 were similar to those of SMST1 and SMST2, respectively.

### 4.3. Magnetic Sensitivity Characteristics

**Figure 8:**
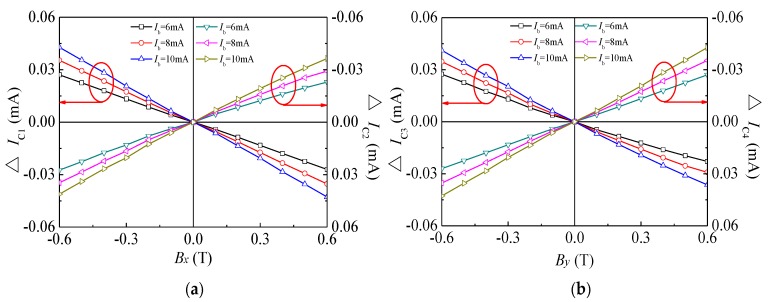
Relationship curves between ∆*I*_C_ and *B* of the four SMSTs: (**a**) SMST1 and SMST2; (**b**) SMST3 and SMST4.

Figure 8 shows the relationship curves between the variation of collector currents for four SMSTs and magnetic field *B_x_* (*B_y_*), where ∆*I_C_*_1_, ∆*I_C_*_2_, ∆*I_C_*_3_ and ∆*I_C_*_4_ are four collector current variations, respectively. At *V*_CE_ = 5.0 V, we analyze the effects of *I_B_* on magnetic sensitivity. Figure 8a clearly reveals that when *B_x_* > 0 T (or *B_x_* < 0 T), ∆*I_C_*_1_ is less than zero (or greater than zero) and ∆*I*_C2_ value is greater than zero (or less than zero). When *B_x_* is a fixed value, the absolute value of ∆*I_C_*_1_ and ∆*I_C_*_2_ increases with *I_B_*. As shown in Figure 8b, under the same conditions, ∆*I_C_*_3_ and ∆*I_C_*_4_ are similar to those of ∆*I_C_*_1_ and ∆*I_C_*_2_, respectively. The experimental results showed that *V*_CE_ and *B_x_* (*B_y_*) were fixed values, the absolute value of ∆*I_C_*_1_, ∆*I_C_*_2_, ∆*I_C_*_3_ and ∆*I_C_*_4_ linearly increasing with *I_B_*.

**Figure 9:**
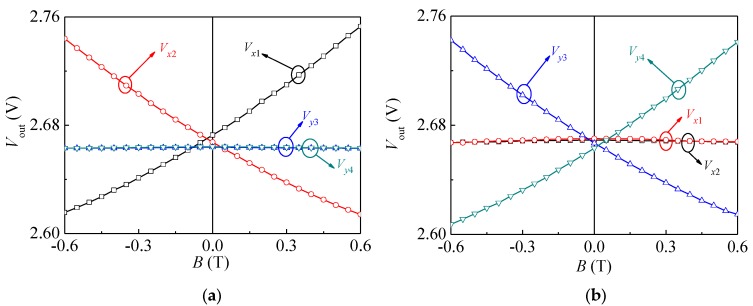
Relationship curves between *V*_out_ and *B* of the SMSTs: (**a**) *B* = *B_x_*; (**b**) *B* = *B_y_*.

In light of Figure 2, the input-output characteristics of the SMSTs tested under *V*_CE_ = 5.0 V and *I_B_* = 8.0 mA are shown in Figure 9. When *B* = *B_x_*, it can be seen from Figure 9a that when *B* > 0 T (or *B* < 0 T), *V_x_*_1_ increases with *B* and *V_x_*_2_ decreases with *B* (or *V_x_*_1_ decreases with *B* and *V_x_*_2_ increases with *B*). The experimental results showed that SMST1 and SMST2 had the opposite magnetic field sensitive directions, while *V_y_*_3_ and *V_y_*_4_ were almost unchanged, indicating that the magnetic field applied in the direction of the *x*-axis had little effect on SMST3 and SMST4.

Moreover, under the same conditions, when the magnetic field was applied along the *y*-axis direction, the collector output voltage of SMST3 and SMST4 were similar to those of SMST1 and SMST2, respectively. As a result, Figure 9b reveals that when the magnetic field was applied along the *y*-axis, it only affected SMST3 and SMST4 and had the opposite sensitive direction. In line with Equation (4), the voltage magnetic sensitivities (*S_x_*_1_, *S_x_*_2_, *S_y_*_3_ and *S_y_*_4_) of the four SMSTs can be calculated from the experimental results. When *V*_CE_ = 5.0 V and *I_B_* = 8.0 mA, *S_x_*_1_, *S_x_*_2_, *S_y_*_3_ and *S_y_*_4_ are 115 mV/T, 108 mV/T, 106 mV/T and 112 mV/T, respectively.

**Figure 10:**
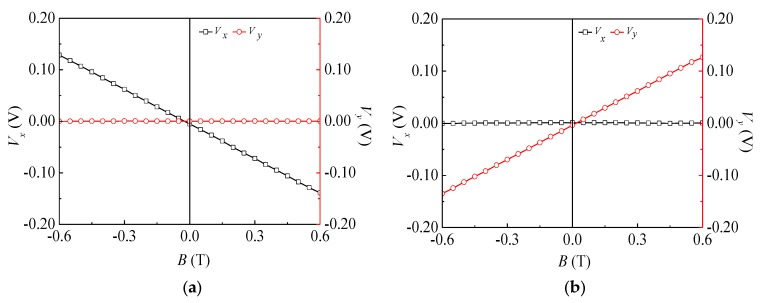
Relationship curves of *V_x_*~*B*, *V_y_*~*B* of the 2D magnetic field sensor: (**a**) *B* = *B_x_*; (**b**) *B* = *B_y_*.

The relationship curves between *V_x_* (*V_y_*) of the *x*-axis and *y*-axis sensors and *B* are shown in Figure 10, which shows that *V_x_* and *V_y_* had better linearity with changing *B,* and the proposed sensor could detect the 2D magnetic field. As obtained from Equation (4), *S_xx_* and *S_yy_* of DSІ and DSII were 223 mV/T and 218 mV/T, respectively. Based on Equation (5), *S_x__y_* and *S_y__x_* of DSІ and DSII were 0.43 mV/T and 0.08 mV/T, respectively. On the basis of the definition of cross interference [13], the cross interference was found to be 0.19% and 0.04%, respectively. The results showed that the sensitivities of the sensor could be improved with better uniformity and lower cross interference.

### 4.4. Characteristics of the 2D Magnetic Field Sensor

**Figure 11:**
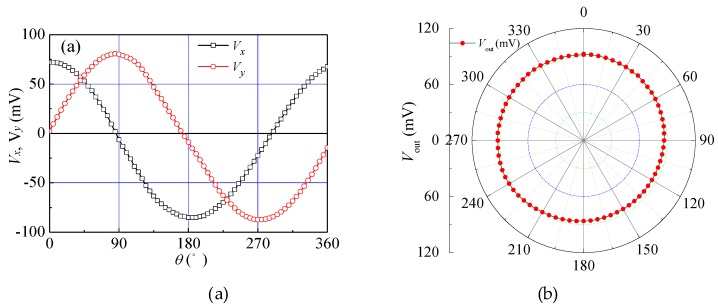
Relationship curves between the output voltage and rotation angle *θ* of the proposed sensor: (**a**) *V_x_*~*θ* and *V_y_*~*θ*; (**b**) *V*_out_~*θ*.

When *V*_DD_ = 5.0 V and *I*_B_ = 8.0 mA, Figure 11a,b shows the relationship curves between *V_x_* (*V_y_*) and rotation angle *θ* (from 0° to 360° with a step of 5°). Under the condition of *B* = 0.4 T, we rotated the chip, when *θ* = 0° (*B* = *B_x_* and *B_y_* = 0 T), *V_x_* was maximum and *V_y_* was approximately zero. With the increasing of *θ*, *V_y_* increased and *V_x_* decreased. When *θ* = 45°, in theory, $B_{x}=B_{y}=\sqrt{2}B/2$, the experimental results showed that *V_x_* = 53.1 mV and *V_y_* = 58.9 mV. When *θ* = 90°, the experimental results showed that *V_x_* = −6.5 mV and *V_y_* = 80.1 mV. From Figure 11a, we can see that *V_x_* and *V_y_* varied with *θ* and conformed to the sine and cosine functional relationship. Through the analysis of the experimental results, the proposed sensor could detect *B_x_* and *B_y_* in the *xy* plane, respectively. According to Equation (6), we calculated the *V*_out_ of the sensor at different rotation angles. Figure 11b shows the relationship curves between *V*_out_ and *θ*, and *V*_out_ was close to the circular. The results showed that the magnetic sensitivities along the *x*-axis and *y*-axis direction of the 2D magnetic field sensor approached uniformity.

Table 1 gives a summary of the performance of the 2D magnetic field sensors [9,10,11,12]. In this work, the monolithically-integrated magnetic field sensor based on four SMSTs and four collector load resistors had higher magnetic sensitivity and lower cross interference.

## 5. Conclusions

In summary, a monolithically-integrated 2D magnetic field sensor was designed and fabricated on a silicon wafer with the <100> direction (*ρ* > 100 Ω·cm) using MEMS technology and packaged on PCBs. It consisted of two difference structures with four SMSTs and four collector load resistors. When *V*_CE_ = 5.0 V and *I*_B_ = 8.0 mA, the magnetic sensitivities of the two difference structures for the proposed sensor were 223 mV/T and 218 mV/T, respectively. The *S_xx_* and *S_yy_* were approximately equal, indicating that the magnetic sensitivity of the proposed sensor had better uniformity. Through the characteristics analysis of the proposed sensor, the relationship curves between output voltage (*V_x_*, *V_y_*) and *θ* conformed to the sine function and cosine function at a constant external magnetic field (*B*), so the proposed sensor could detect the magnetic field vector ***B*** in the *xy* plane and exhibited lower cross interference, which lays the foundation for the research on the measurement of a 2D magnetic field and for the monolithic integration of the sensor.

## Figures and Tables

**Table 1:** Performance summary and comparison of 2D magnetic field sensors.

Reference Parameters	Magnetic Sensitive Structure	Supply Voltage	Magnetic Sensitivity	Cross Interference	Chip Area
[9]	Hall device	2.7 V	9.56 mV/T	0.259 mV (*B* = 150 mT) 0.330 mV (*B* = 150 mT)	2.0 × 1.0 mm^2^
[10]	Vertical Hall device	—	*S_x_* = 40.06 mV/(V·T) *S_y_* = 42.65 mV/(V·T)	12.55 mV/(V·T) 12.33 mV/(V·T)	—
[11]	Hall device	5.0 V	34.0 mV/(V·T)	—	60.0 × 60.0 μm^2^
[12]	Magnetic sensitive transistor	*V*_CE_ = 10.0 V *I_B_* = 6.0 mA	*S_x_* = 366 mV/T *S_y_* = 365 mV/T	—	7.0 × 7.0 mm^2^
This work	Magnetic sensitive transistor	*V*_CE_ = 5.0 V *I_B_* = 8.0 mA	*S_x_* = 223 mV/T *S_y_* = 218 mV/T	0.19% 0.04%	2.3 × 2.3 mm^2^
